# Dietary Intake of Red Meat, Processed Meat, and Poultry and Risk of Colorectal Cancer and All-Cause Mortality in the Context of Dietary Guideline Compliance †

**DOI:** 10.3390/nu13010032

**Published:** 2020-12-23

**Authors:** Heddie Mejborn, Sanne Pagh Møller, Lau Caspar Thygesen, Anja Biltoft-Jensen

**Affiliations:** 1 National Food Institute, Technical University of Denmark, 2800 Kgs. Lyngby, Denmark; apbj@food.dtu.dk; 2 National Institute of Public Health, University of Southern Denmark, 1455 Copenhagen K, Denmark; sapm@sdu.dk (S.P.M.); lct@sdu.dk (L.C.T.)

**Keywords:** prospective cohort study, colorectal cancer, all-cause mortality, meat, dietary quality

## Abstract

Meat intake has been linked to increased risk of colorectal cancer (CRC) and mortality. However, diet composition may affect the risks. We aimed to estimate associations between red and processed meat and poultry intake and risk of CRC and all-cause mortality and if they are modified by dietary quality using Cox regression analyses. Baseline dietary data were obtained from three survey rounds of the Danish National Survey on Diet and Physical Activity. Data on CRC and all-cause mortality were extracted from national registers. The cohort was followed from date of survey interview—or for CRC, from age 50 years, whichever came last, until 31 December 2017. Meat intake was analysed categorically and continuously, and stratified by dietary quality for 15–75-year-old Danes at baseline, n 6282 for CRC and n 9848 for mortality analyses. We found no significant association between red and processed meat intake and CRC risk. For poultry, increased CRC risk for high versus low intake (HR 1.62; 95%CI 1.13–2.31) was found, but not when examining risk change per 100 g increased intake. We showed no association between meat intake and all-cause mortality. The association between meat intake and CRC or mortality risk was not modified by dietary quality.

## 1. Introduction

In October 2015, the International Agency for Research on Cancer concluded that processed meat could be classified as “carcinogenic to humans”, and red meat could be classified as “probably carcinogenic to humans” [1,2]. The conclusions were primarily related to colorectal cancer (CRC). In 2018, the World Cancer Research Fund and American Institute for Cancer Research stated that there is strong evidence that consuming red and processed meat increases the risk of CRC [3]. Poultry is not mentioned as a risk factor for CRC, neither by the International Agency for Research on Cancer nor by the World Cancer Research Fund and American Institute for Cancer Research.

Some, but not all, prospective, population-based studies from different countries have associated high red and processed meat intake with increased risk of mortality, in particular in American cohorts [4,5,6,7,8]. An inverse association between poultry intake and total mortality was observed among low meat consumers in Asia [8] and among American men [4]. Thus, a high intake of red and processed meat but not poultry seems to be associated with CRC, and in some populations with mortality.

Since composition of diets is complex and persons with different diets may differ on other characteristics, cohort studies on associations between meat intake and health have many confounders. However, it is possible to have a high meat content in a healthy diet [9]. Therefore, we suggest that analyses of associations between meat intake and disease risk should be stratified by dietary quality. Dietary quality should be expressed as a diet quality index and not as division in, e.g., “Western” and “Mediterranean” diets, where a high meat intake automatically becomes a proxy for an unhealthy diet, and where it is not possible to correct for all the dietary confounders, of which several are inter-correlated.

The aim of the study is to evaluate the associations between the intake of red and processed meat and poultry and CRC and all-cause mortality risk, both in an adult study population in general and in subgroups with different dietary guideline compliance, minimising the influence of diet-related confounding.

## 2. Materials and Methods

### 2.1. Definitions

We defined red meat as unprocessed muscle tissue from mammals such as beef, veal, pork, and lamb. A small intake of unprocessed edible offal, e.g., liver and heart was included. The meat could be minced and/or frozen. It was usually eaten cooked.

Processed meat was red meat or poultry that undergoes a transformation and contains approved ingredients and may be subject to some form of preservation; in other words: smoking, drying, curing or fermentation.

Poultry included meat from chicken, hen, turkey, goose, dove, duck, and pheasant. However, only the intake of chicken and turkey was at a sizable level among Danes.

Values for meat intake were expressed as cooked meat.

### 2.2. Diet Information and Study Population

The analyses were based on information from adults, who participated in The Danish National Survey on Diet and Physical Activity in any of the three survey rounds (year 2000–2002, 2003–2008, or 2011–2013). Invited individuals were randomly drawn from the Danish Civil Registration System and comprised non-institutionalised free-living Danish citizens [10]. Data for food intake were obtained via self-administered, quantitative seven-day pre-coded food diaries [11]. Information on intake of meat (red meat, processed meat, and poultry), dietary guideline compliance, energy intake, alcohol energy intake, body mass index (BMI), smoking habits, and leisure physical activity were extracted from the surveys. For participants who had answered more than one survey (n = 89), information from the first survey was included. Thus, diet information was only measured once for each participant. In total, 9848 individual participants were 15–75 year of age at baseline and were therefore eligible for inclusion in the study. Participants with cancers other than CRC were not excluded from the study population, and we did not censor for other cancers during follow-up.

The diet of each participant was assigned a Dietary Guideline Compliance Score (DGCS) based on how well the diet complies with the five quantitative official Danish dietary guidelines. The recommended dietary contents are 600 g fruit and vegetables/10 MJ, 350 g fish/10 MJ, 75 g whole grain/10 MJ, saturated fatty acids max 10% of total energy intake (E%), and added sugars max 10 E%. For each dietary guideline, a score of 0–1 was given by dividing the dietary content of the actual component with the recommended dietary content (scores > 1 were truncated at 1). The five scores were added to yield the total score between 0 and 5. We designate participants with a low DGCS (<3.1) as low-compliers and participants with a high DGCS (≥3.1) as high-compliers in analyses of the CRC cohort (see description of the cohort below). In the all-cause mortality cohort, low, medium, and high DGCS were <2.4, 2.4–3.7 and >3.7, respectively.

### 2.3. Register-Based Information

Outcomes were identified through linkage of the study population with information from registers using the unique personal identification number [12]. Information on incident CRC was based on histologically confirmed cancer from the Danish Cancer Registry (ICD-10: C18 and C20) [13]. The Danish Cancer Registry includes information on all diagnosed cases of cancer in Denmark. Only information about incident CRC was retrieved for this study. Information on all-cause mortality was based on information on date of death regardless of underlying cause from the Register of Causes of Death [14].

Information on age, sex, ethnicity, and emigration were obtained from the Danish Civil Registration System [12]. Educational attainment (short = primary school, medium = high school or vocational school, long = higher education) was based on the Population’s Educational Register [15].

From information on primary diagnosis in the National Patient Register, colorectal polyps up to 10 years before baseline (ICD-10: K62.1 and K63.5) were identified. Screening for colorectal cancer was not introduced in Denmark until 2014, and information on colorectal polyps was so rare in the study population (n = 5) that they were not included as a confounder in the analyses.

### 2.4. Analyses

In analyses of CRC, the aim was to study incident cases of disease, and, therefore, individuals were excluded if they had been diagnosed with CRC before baseline (n = 31). In addition, participants younger than 50 years of age before end of follow-up were excluded (n = 3535), as CRC was almost absent among this part of the study population. The CRC cohort (n = 6282) was followed from baseline (date of survey interview) or from age 50 years for those not 50 years old at baseline (delayed entry). Follow-up ended at first CRC event (first diagnosis or death due to CRC) or at emigration, death due to other causes or end of follow-up (31 December 2017), whichever came first. The mean time of follow-up was 8.7 years. For analyses of all-cause mortality, the cohort (n = 9848) was followed from baseline, and follow-up ended at death, emigration, or end of follow-up (31 December 2017), whichever came first. The mean time of follow-up was 10.8 years. In both analyses, there was no loss to follow-up.

Missing information on country of origin (0.01%) was imputed as Danish origin, missing educational level (1.5%) was imputed as short education, missing BMI (1.0%) was imputed as normal BMI (18.5–25), missing smoking status (1.1%) was imputed as never smoker, and missing information on physical activity (0.4%) was imputed as the most common category (moderate/hard).

### 2.5. Meat Intake and Dietary Guideline Compliance

We use the designation “categorical” when we compare groups with different intake, while we use “continuous” for analyses on increments per 50 or 100 g/day. Intake of red meat, processed meat, and poultry was analysed on both a continuous and categorical scale. For analyses of all-cause mortality, the measures of meat intake were categorised in three groups (lower quartile; the two middle quartiles together; upper quartile). Due to the small number of CRC cases in some groups, it was not possible to perform the statistical analyses with such categorisation. Therefore, meat intake was categorised in two groups (below median; median and above) in CRC analyses. The intake of different types of meat (mean, SD, median) was almost identical in the CRC cohort and all-cause mortality cohort (data not shown). Therefore, and to eliminate categorisation of meat intake as reason for the differences in the analyses, we used intake in the all-cause mortality cohort to categorise meat intake in both CRC and all-cause mortality analyses. For analyses of all-cause mortality and CRC, the measures of dietary guideline compliance (DGC) were categorised in a similar way as meat intake. For analyses of associations between meat intake as a continuous variable and outcomes, red meat, and poultry were expressed per 100 g increments per day, and processed meat per 50 g increments per day.

### 2.6. Associations between Meat Intake and Dietary Guideline Compliance and Outcomes

The associations between meat intake and outcomes were estimated as hazard ratios (HRs) with 95% confidence intervals (CIs) using Cox regression analyses. As all studied outcomes were strongly associated with age, we used age as the underlying timescale in the analyses. Different measures of meat intake were included in different regression models with adjustment for sex, educational attainment (the year before baseline), ethnicity, smoking, physical activity, alcohol, BMI, and total energy intake. To test if non-linear effects better represented the associations between meat intake and outcomes compared with linear effects, quadratic and cubic terms were included in the regression models. However, all non-linear effects were non-significant, so meat intake was only included linearly. Valid results from Cox regression analyses require that the hazard ratio between groups does not change with age, i.e., the assumption of proportional hazards. To evaluate if the assumption of proportional hazards was fulfilled, we estimated the Schoenfeld residuals for each of exposure variable. We then tested in a linear regression model whether these residuals were correlated with age (underlying timescale). These analyses indicated that the assumption of proportional hazards was fulfilled. We also visually inspected the log-negative-log survival curves for each of the exposure and outcome variables. These plots did not indicate violation of the proportional hazard assumption.

The associations between DGC and the studied outcomes were estimated using the same methods as for meat intake, but DGC was only included categorical.

### 2.7. Associations between Meat Intake and Outcomes Stratified by Dietary Guideline Compliance

To evaluate whether the association between meat intake and disease outcome differed depending on DGC, associations stratified by DGC were estimated using Cox regression analyses with age as the underlying timescale. In these analyses, the statistical significance of an interaction between meat intake and DGC was tested by including both meat intake and DGC as separate main effects and as an interaction term with each other. These tests were performed both on analyses with meat intake as a categorical and a continuous variable.

All analyses were performed using SAS 9.4 (SAS Institute Inc, Cary, CA, USA). A *p*-value <0.05 was considered statistically significant.

## 3. Results

We ascertained 127 incident CRC cases, and 640 persons died during follow-up.

Characteristics of the CRC study population stratified by DGC and red meat intake are shown in Table 1. Characteristics of the study population stratified by DGC and processed meat and poultry intake, respectively, are shown in Supplementary Tables S1 and S2.

In the total study population, 51.7% were women, most had a medium educational level and were of normal weight, more than half of the population were either former or current smokers, and half of the population had a moderate/hard level of physical activity.

Most characteristics seemed to differ when groups with different DGC and meat intake were compared. For example, men made up a large proportion of those with a high meat intake and low compliance with dietary guidelines, whereas the women dominated the low-meat groups. In the groups with high DGC, more participants had a long education, and fewer were current smokers compared with groups with low DGC. Participants in groups with high DGC were more physically active in their leisure time compared with groups with low DGC, but this was not reflected in the weight status of the groups.

The intake distribution of the types of meat analysed is shown in Table 2. The daily median red meat intake was approximately twice as high as the processed meat intake and four times as high as the poultry intake. The 25% of participants with the lowest poultry intake were eating 1 g of poultry per day or less because several participants were not eating poultry during the dietary survey.

### 3.1. Association between Meat Intake and Colorectal Cancer and All-Cause Mortality

No significant associations were found between red meat and processed meat intake and CRC risk (Table 3). High poultry intake, however, increased the CRC risk significantly (HR = 1.62; 95%CI: 1.13–2.31) compared with low poultry intake, but such increase was not observed when examining risk change per 100 g per day (HR = 1.39; 95%CI: 0.69–2.77; *p* for trend = 0.34).

The total meat intake did not significantly affect CRC risk in our study (data not shown).

No significant associations were found between red meat, processed meat, and poultry intake and all-cause mortality (Table 4).

### 3.2. Association between Dietary Guideline Compliance and Colorectal Cancer and All-Cause Mortality

There was no significant increased CRC risk among those with a low DGC compared with those with a high DGC (HR = 1.09; 95%CI: 0.75–1.58; *p* for trend = 0.66) (Table 5).

DGC did not affect all-cause mortality risk (HR = 1.26; 95%CI:0.99–1.61; *p* for trend = 0.13) (Table 6).

### 3.3. Association between Meat Intake and Colorectal Cancer and All-Cause Mortality, Stratified by Dietary Guideline Compliance

The associations between meat intake and CRC stratified by DGC are shown in Table 7. For red meat, the CRC risk for high versus low intake and per 100 g per day was not affected by DGC (*p* for interaction = 0.53 and *p* for interaction = 0.45, respectively). Similarly, the CRC risk for high versus low processed meat intake and per 50 g processed meat per day was not affected by DGC (*p* for interaction = 0.47 and *p* for interaction = 0.97, respectively). DGC was not found to significantly modify the association between poultry intake and CRC risk (*p* for interaction = 0.75 for high versus low poultry intake and *p* for interaction = 0.89 per 100 g per day, respectively).

There was no significant association between meat intake and all-cause mortality stratified by DGC for any of the meat types (Table 8). For red meat, DGC neither affected all-cause mortality risk for high versus low intake nor per 100 g per day (*p* for interaction = 0.98 and *p* for interaction = 0.85, respectively). Similarly, for high versus low intake and per 50 g per day of processed meat, DGC did not affect all-cause mortality risk (*p* for interaction = 0.65 and *p* for interaction = 0.28, respectively). For poultry, DGC did not affect the risk of all-cause mortality for high versus low intake (*p* for interaction = 0.88), or per 100 g per day (*p* for interaction = 0.21).

## 4. Discussion

### 4.1. Colorectal Cancer

We found no significant association between red meat or processed meat intake and CRC risk. For poultry, however, high compared with low intake increased CRC risk significantly by 62%, but we found no increased CRC risk per 100 g poultry per day.

In accordance with our result on red meat, several prospective cohort studies representing more than 10 European countries, including Denmark, found no association between red meat intake and CRC risk [16,17,18,19], but an increased hazard ratio of CRC per 1 serving of red meat per day was seen in two American prospective cohorts [20]. In meta-analyses covering America, Australia, European and Asian countries, a positive association between red meat intake and CRC risk has been observed [19,21,22]. The association was stronger in Asian and Australian cohorts compared with European and North American cohorts [21]. The latest meta-analysis performed by the World Cancer Research Fund Continuous Update Project found that red meat intake was positively associated with CRC risk [23].

It is difficult to obtain and compare information about actual meat intake (g/day) for different types of meat in different cohorts, which affects the possibility to compare outcomes in studies of effects of high versus low meat intake. For example, high red meat intake in an Asian cohort may be similar in magnitude to low red meat intake in some Western cohorts.

In contrast to our results on processed meat, others found positive associations between processed meat intake and CRC risk in American, Australian, Asian, and European cohorts [16,17,20,21,22,23]. However, no association was found in the Danish Diet, Cancer and Health cohort study [18].

Studies on the effects of meat intake from different countries and continents can be difficult to compare because the proportions of the different types of red and processed meat differ significantly between regions. The types of meat—both red and processed meat—constitute different hazards due to their structure and composition. Moreover, certain meat subtypes may be more prevailing in unhealthy diets than others, which can affect the risk estimates. Therefore, analyses on effects of meat subtypes can contribute to our understanding of differences observed in different cohorts and are warranted in future studies.

In a meta-analysis comparing the highest versus lowest red meat intake in Asian and European cohorts, Carr et al. [24] found that beef intake was associated with an increased risk of CRC in European cohorts but no association was found for pork. In a Danish cohort, no associations were seen for beef or pork intake and colon cancer risk but beef intake was associated with decreased risk and pork intake with increased risk of rectal cancer [18]. We had too few cases to make subgroup analysis on red meat intake, but from analysis of dietary patterns among the participants [25], we know that pork constitutes a slightly higher part of their red meat intake than beef/veal, which may have affected our findings.

For poultry intake, our results were in contrast with what others have found. No association between poultry intake and CRC risk was reported by the World Cancer Research Fund Continuous Update Project [23] or seen in European cohorts [16,17,18]. A decreased CRC risk was associated with 50 g poultry increment per day in a meta-analysis including prospective cohort studies from America, Australia, Europe, and Japan [26]. Thus, more studies are needed to confirm our findings.

A pronounced difference in meat content in high-meat diets with different healthy eating indices was found by Kappeler et al. [4]. Thus, comparing groups with low and high meat intake without considering dietary quality and what foods replace the meat will simultaneously be a comparison of healthy and unhealthy diets. Therefore, we analysed our data by looking at the effects of meat intake stratified by DGCS to reduce the confounding from dietary quality. However, when stratified by DGC, we found no statistically significant differences in the associations between meat intake and CRC risk in low-compliers and high-compliers.

Norat et al. [16] found that the CRC risk associated with high intakes of red and processed meat was more pronounced in participants from a European cohort including Denmark with low and medium fibre intake (≤26–28 g/day) compared with those with high fibre intake (>26–28 g/day). Others have found that in two US cohorts, an increase in total fibre, cereal fibre, or whole-grain intake of 5 g per day reduced CRC risk by 7–25%, while fibres from fruit and vegetables did not have such effect [27]. From dietary pattern analyses of our participants’ diet, we know that those who comply well with dietary guidelines had both a high whole-grain intake and total fibre intake, but it apparently did not influence the CRC risk associated with meat intake.

### 4.2. All-Cause Mortality

We found no significant associations between red meat, processed meat, and poultry intake and all-cause mortality.

Similar results were found for red meat in a large American cohort [4] but not in another American cohort [6], and not in European cohorts [6,28]. Three meta-analyses showed no associations between red meat intake and all-cause mortality risk [5,6,29], while one meta-analysis showed that each additional intake of 100 g red meat/day was positively associated with all-cause mortality [30].

In contrast to our results, in a European cohort including Denmark, intake of processed meat was positively associated with all-cause mortality [28], which was also the result of four meta-analyses [5,6,29,30].

In a recent meta-analysis, Han et al. found a small, positive association between red and processed meat intake and cancer mortality, but the evidence was rated to be of low certainty [31].

White meat (including chicken, turkey, and rabbit) intake was not associated with all-cause mortality in meta-analyses of prospective cohort studies [5,28]. Likewise, no association was found between poultry intake and CRC mortality in a dose-response meta-analysis of prospective cohort studies [26], and no association was found between poultry intake and cancer mortality in a meta-analysis of prospective cohort studies [32].

In our study, a diet composition that did not comply well with the official, quantitative Danish dietary guidelines (independent of meat content) was significantly associated with mortality risk in the least adjusted model (adjusted by sex and age) (HR 1.66; 95%CI 1.32–2.10). However, DGCS was not significantly associated with mortality risk in the multivariate model (adjusted by sex, age, educational attainment, ethnicity, smoking, physical activity, alcohol, BMI, and total energy intake), and *p* for trend showed no significant effect of DGCS.

Kappeler et al. [4] found a 27% decreased mortality risk among Americans with the top third Healthy Eating Index score (developed by the US Department of Agriculture) compared with the bottom third Healthy Eating Index score. Unfortunately, these authors did not estimate the all-cause mortality risk in participants with different meat intake stratified by dietary quality.

### 4.3. Strengths and Limitations

Our study had several strengths. The studied outcomes were based on national registers with high validity and completeness, and we included complete information on migration and death ensuring complete follow-up of the study cohort. The linkage also enabled us to include only incident cases of disease and to minimise the risk of reverse causality as we excluded those with disease before baseline. The study included comprehensive information on dietary components, which made it possible to evaluate if associations differed with DGC. The diet registration for each participant included seven days including weekend days, and the data collection process in the study population covered all seasons to allow for seasonal variations in dietary data on study population basis.

However, the study also had limitations. The dietary surveys were representative regarding gender and age. However, in the latest surveys, participants with short education were under-represented, which may limit the generalisability of the findings. In addition, the study only included one dietary registration for each individual. Therefore, it was assumed that the diet composition did not change during follow-up, but if the population had large variations in DGC during follow-up, this would influence the estimated associations. Finally, as mentioned previously, the size of the study population affected the power to identify statistically significant associations, especially in analyses on interactions between meat intake and DGC, where the numbers of participants in groups were low.

In the analyses, BMI was included as a confounder, as is common practice in similar studies. However, it is likely that BMI is a mediator instead of a confounder in the presented associations, implying that the presented results have been over-adjusted. Analyses without BMI in the model (data not shown) showed that inclusion of BMI only mildly attenuated the estimates, and that the results on low DGCS did not become significant when BMI was not included in the model.

We did not find statistically significant associations between meat intake and CRC or mortality risk. However, the ability to reach statistically significant results is influenced by many factors. For example, since the study population was 15–75 years at baseline, a large proportion of the population was too young to be at real risk of developing CRC. This is why we only studied CRC risk among individuals aged 50 years and older. Thus, the number of outcomes could be an explanation why the associations between meat intake and the CRC risk was non-significant. Similarly, a large proportion of the population were too young to be at an appreciable mortality risk.

Analyses of dietary patterns in our cohort showed that a low dietary content of one type of meat, e.g., poultry, was associated with a high dietary content of other types of meat, e.g., red meat [25]. Thus, dietary content of meat types could be confounders. Before we made the estimates of associations between meat intake and disease risk, it was not known to us exactly which types of dietary meat content were associated, and, therefore, we did not include different types of meat in the same analyses. However, in future analyses, it may be appropriate to take dietary content of other types of meat or other replacement foods into consideration.

Another limitation was that the size of the study population restricted our opportunity to study differences between those with very low and those with a very high meat intake. In analyses of CRC risk, we were only able to divide the population’s meat intake into two groups instead of quartiles. This introduced some arbitrariness around cut-off values of meat intake since we split the population into two groups without having a meaningful difference for meat consumed around the median. However, in the interpretation of results, we also focused on estimates of associations with meat intake on a continuous scale, which did not suffer from this limitation.

## 5. Conclusions

We showed no significant associations between red and processed meat intake and risk of CRC and all-cause mortality. A significant increase in CRC risk, but not in all-cause mortality, was found for high versus low poultry intake but not for risk change per 100 g increment per day. None of these associations were modified by dietary guideline compliance.

## Figures and Tables

**Table 1:** Baseline characteristics of the colorectal cancer study population stratified by dietary guideline compliance and intake of red meat, n = 6282.

Dietary Guideline Compliance ^1^		Low		High	
Red Meat Intake ^2^		Low	High	Low	High
Age, mean (SD)	54 (11)	52 (11)	52 (11)	55 (11)	56 (10)
**Total study population**		**n (%)**	**n (%)**	**n (%)**	**n (%)**
Sex					
Men	3033 (48.3)	502 (44.2)	1164 (68.4)	563 (29.2)	804 (53.1)
Women	3249 (51.7)	635 (55.8)	538 (31.6)	1365 (70.8)	711 (46.9)
Ethnicity					
Danish	6128 (97.5)	1098 (96.6)	1682 (98.8)	1873 (97.1)	1475 (97.4)
Western	79 (1.3)	24 (2.1)	7 (0.4)	33 (1.7)	15 (1.0)
Non-Western	75 (1.2)	15 (1.3)	13 (0.8)	22 (1.1)	25 (1.7)
Educational level^3^					
Long	1927 (30.7)	276 (24.3)	389 (22.9)	724 (37.6)	538 (35.5)
Medium	2665 (42.4)	519 (45.6)	817 (48.0)	719 (37.3)	610 (40.3)
Short	1690 (26.9)	342 (30.1)	496 (29.1)	485 (25.2)	367 (24.2)
BMI					
Underweight	91 (1.4)	23 (2.0)	24 (1.4)	29 (1.5)	15 (1.0)
Normal weight	3121 (49.7)	598 (52.6)	755 (44.4)	1049 (54.4)	719 (47.5)
Overweight	2251 (35.8)	373 (32.8)	680 (40.0)	634 (32.9)	564 (37.2)
Obese	819 (13.0)	143 (12.6)	243 (14.3)	216 (11.2)	217 (14.3)
Smoking					
Never	2598 (41.4)	397 (34.9)	598 (35.1)	905 (46.9)	698 (46.1)
Former	1959 (31.2)	290 (25.5)	468 (27.5)	644 (33.4)	557 (36.8)
Current	1725 (27.5)	450 (39.6)	636 (37.4)	379 (19.7)	260 (17.2)
Leisure time physical activity					
None	520 (8.3)	140 (12.3)	185 (10.9)	117 (6.1)	78 (5.1)
Light	2562 (40.8)	497 (43.7)	721 (42.4)	777 (40.3)	567 (37.4)
Moderate/hard	3200 (50.9)	500 (44.0)	796 (46.8)	1034 (53.6)	870 (57.4)

Abbreviations: n, number of participants; SD, standard deviation; BMI, body mass index.^1^ Low compliance < 3.1 on the Dietary Guideline Compliance Score; high compliance ≥ 3.1 on the Dietary Guideline Compliance Score. Dietary Guideline Compliance Score expresses the dietary compliance with the five quantitative Danish dietary guidelines on fruit and vegetables, fish, whole grain, saturated fatty acids, and added sugars. It can vary between 0 and 5. ^2^ Red meat intake: low < 65 g/day; high ≥ 65 g/day. ^3^ Long: higher education, medium: high school or vocational school, short: primary school.

**Table 2:** Distribution of meat intake (g/day) in the total study population, n = 9848.

Meat Type	Mean	SD	25-Percentile	Median	75-Percentile
Red meat ^1^	75	50	41	65	97
Processed meat ^2^	43	35	19	35	58
Poultry ^3^	23	27	1	16	34

Abbreviations: SD, standard deviation. ^1^ Unprocessed muscle tissue from beef, veal, pork, and lamb, including a small amount of unprocessed edible offal. ^2^ Red meat or poultry that contains approved ingredients and may be subject to some form of preservation. ^3^ Mainly chicken and turkey.

**Table 3:** Association between intake of different types of meat and risk of colorectal cancer, n = 6282.

Meat Intake	No. of Cases	IR ^1^	HR (95%CI) ^2^	HR (95%CI) ^3^	*p*-Value for Trend
Red meat ^4^					
Low	64	228	1.00 Reference	1.00 Reference	
High	63	235	1.00 (0.70;1.44)	1.01 (0.69;1.48)	
Per 100 g/day			1.04 (0.69;1.56)	1.04 (0.67;1.61)	0.86
Processed meat ^5^					
Low	65	225	1.00 Reference	1.00 Reference	
High	62	238	1.07 (0.74;1.55)	1.10 (0.74;1.63)	
Per 50 g/day			1.14 (0.86;1.51)	1.16 (0.85;1.59)	0.34
Poultry ^6^					
Low	53	189	1.00 Reference	1.00 Reference	
High	74	275	1.60 (1.12;2.28)	1.62 (1.13;2.31)	
Per 100 g/day			1.37 (0.68;2.73)	1.39 (0.69;2.77)	0.34

Abbreviations: n, number of participants; IR, incidence rates; HR, hazard ratios; CI, confidence interval. ^1^ Per 100,000 person-years. ^2^ Adjusted by sex. ^3^ Adjusted by sex, educational attainment, ethnicity, smoking, physical activity, alcohol, BMI, and total energy intake. ^4^ Red meat intake: low < 65 g/day; high ≥ 65 g/day. ^5^ Processed meat intake: low < 35 g/day; high ≥ 35 g/day. ^6^ Poultry intake: low < 16 g/day; high ≥ 16 g/day.

**Table 4:** Association between intake of different types of meat and risk of all-cause mortality, n = 9848.

Meat Intake	No. of Cases	IR^1^	HR (95%CI) ^2^	HR (95%CI) ^3^	*p*-Value for Trend
Red meat ^4^					
Low	167	602	1.00 Reference	1.00 Reference	
Medium	356	650	0.95 (0.79;1.14)	1.02 (0.84;1.23)	
High	117	493	0.77 (0.60;0.98)	0.86 (0.67;1.12)	
Per 100 g/day			0.81 (0.67;0.98)	0.89 (0.72;1.09)	0.26
Processed meat ^5^					
Low	180	702	1.00 Reference	1.00 Reference	
Medium	328	601	0.89 (0.73;1.07)	1.04 (0.80;1.36)	
High	132	506	0.88 (0.69;1.12)	1.02 (0.82;1.26)	
Per 50 g/day			0.95 (0.83;1.08)	0.99 (0.85;1.15)	0.92
Poultry ^6^					
Low	225	852	1.00 Reference	1.00 Reference	
Medium	277	526	0.87 (0.73;1.04)	0.98 (0.82;1.17)	
High	138	509	0.85 (0.69;1.06)	0.92 (0.74;1.14)	
Per 100 g/day			0.81 (0.58;1.14)	0.91 (0.65;1.28)	0.59

Abbreviations: n, number of participants; IR, incidence rates; HR, hazard ratios; CI, confidence interval. ^1^ Per 100,000 person-years. ^2^ Adjusted by sex. ^3^ Adjusted by sex, educational attainment, ethnicity, smoking, physical activity, alcohol, BMI, and total energy intake. ^4^ Red meat intake: low < 41 g/day; medium 41–97 g/day; high > 97 g/day. ^5^ Processed meat intake: low < 19 g/day; medium 19–58 g/day; high > 58 g/day. ^6^ Poultry intake: low < 1 g/day; medium 1–34 g/day; high > 34 g/day.

**Table 5:** Association between dietary guideline compliance and risk of colorectal cancer, n = 6282.

Dietary Guideline Compliance ^1^	No. of Cases	IR ^2^	HR (95%CI) ^3^	HR (95%CI) ^4^	*p*-Value for Trend
Low	61	242	1.17 (0.82;1.67)	1.09 (0.75;1.58)	
High	66	223	1.00 Reference	1.00 Reference	0.66

Abbreviations: n, number of participants; IR, incidence rates; HR, hazard ratios; CI, confidence interval. ^1^ Low compliance < 3.1 on the Dietary Guideline Compliance Score; high compliance ≥ 3.1 on the Dietary Guideline Compliance Score. Dietary Guideline Compliance Score expresses the dietary compliance with the five quantitative Danish dietary guidelines on fruit and vegetables, fish, whole grain, saturated fatty acids, and added sugars. It can vary between 0 and 5. ^2^ Per 100,000 person-years. ^3^ Adjusted by sex and age. ^4^ Adjusted by sex, age, educational attainment, ethnicity, smoking, physical activity, alcohol, BMI, and total energy intake.

**Table 6:** Association between dietary guideline compliance and risk of all-cause mortality, n = 9848.

Dietary Guideline Compliance ^1^	No. of Cases	IR ^2^	HR (95%CI) ^3^	HR (95%CI) ^4^	*p*-Value for Trend
Low	172	591	1.66 (1.32;2.10)	1.26 (0.99;1.61)	
Medium	331	614	1.19 (0.97;1.45)	1.07 (0.87;1.31)	
High	137	589	1.00 Reference	1.00 Reference	0.13

Abbreviations: n, number of participants; IR, incidence rates; HR, hazard ratios; CI, confidence interval. ^1^ Low compliance < 2.4 on the Dietary Guideline Compliance Score; medium compliance 2.4–3.7 on the Dietary Guideline Compliance Score; high compliance > 3.7 on the Dietary Guideline Compliance Score. Dietary Guideline Compliance Score expresses the dietary compliance with the five quantitative Danish dietary guidelines on fruit and vegetables, fish, whole grain, saturated fatty acids, and added sugars. It can vary between 0 and 5. ^2^ Per 100,000 person-years. ^3^ Adjusted by sex and age. ^4^ Adjusted by sex, age, educational attainment, ethnicity, smoking, physical activity, alcohol, BMI, and total energy intake.

**Table 7:** Association between intake of different types of meat and risk of colorectal cancer. Stratified by dietary guideline compliance, n = 6282.

Dietary Guideline Compliance ^1^	Low, No. of Cases	High, No. of Cases	Low, IR ^2^	High, IR ^2^	Low, HR (95%CI) ^3^	High, HR (95%CI) ^3^	*p*-Value for Interactions
Meat Intake							
Red meat ^4^							
Low	27	37	259	209	1.00 Reference	1.00 Reference	0.53
High	34	29	230	242	0.95 (0.55;1.64)	1.06 (0.62;1.81)	
Per 100 g/day					0.94 (0.51;1.73)	1.12 (0.59;2.12)	0.45
Processed meat ^5^							
Low	27	38	262	205	1.00 Reference	1.00 Reference	0.47
High	34	28	227	252	0.97 (0.55;1.70)	1.22 (0.70;2.12)	
Per 50 g/day					1.24 (0.83;1.86)	1.03 (0.62;1.73)	0.97
Poultry ^6^							
Low	26	27	193	186	1.00 Reference	1.00 Reference	0.75
High	35	39	297	258	1.72 (1.03;2.87)	1.52 (0.93;2.50)	
Per 100 g/day					1.50 (0.56;4.00)	1.29 (0.48;3.48)	0.89

Abbreviations: n, number of participants; IR, incidence rates; HR, hazard ratios; CI, confidence interval. ^1^ Low compliance < 3.1 on the Dietary Guideline Compliance Score; high compliance ≥ 3.1 on the Dietary Guideline Compliance Score. Dietary Guideline Compliance Score expresses the dietary compliance with the five quantitative Danish dietary guidelines on fruit and vegetables, fish, whole grain, saturated fatty acids, and added sugars. It can vary between 0 and 5. ^2^ Per 100,000 person-years. ^3^ Adjusted by sex, age, educational attainment, ethnicity, smoking, physical activity, alcohol, BMI, and total energy intake. ^4^ Red meat intake: low < 65 g/day; high ≥ 65 g/day. ^5^ Processed meat intake: low < 35 g/day; high ≥ 35 g/day. ^6^ Poultry intake: low < 16 g/day; high ≥ 16 g/day.

**Table 8:** Association between intake of different types of meat and risk of all-cause mortality. Stratified by dietary guideline compliance, n = 9848.

Dietary Guideline Compliance ^1^	Low, No. of Cases	Medium, No. of Cases	High, No. of Cases	Low, IR ^2^	Medium, IR ^2^	High, IR ^2^	Low, HR (95%CI) ^3^	Medium, HR (95%CI) ^3^	High, HR (95%CI) ^3^	*p*-Value for Interactions
Meat Intake										
Red meat ^4^										
Low	29	82	56	514	615	637	1.00 Reference	1.00 Reference	1.00 Reference	0.98
Medium	99	191	66	700	654	579	1.18 (0.77;1.80)	1.00 (0.77;1.32)	0.94 (0.65;1.36)	
High	44	58	15	472	511	487	1.07 (0.64;1.76)	0.85 (0.59;1.24)	0.76 (0.41;1.41)	
Per 100 g/day							1.03 (0.73;1.45)	0.87 (0.64;1.18)	0.78 (0.46;1.32)	0.85
Processed meat ^5^										
Low	36	86	58	739	717	662	1.00 Reference	1.00 Reference	1.00 Reference	0.65
Medium	85	176	67	607	611	570	1.12 (0.75;1.70)	0.91 (0.70;1.20)	0.98 (0.67;1.44)	
High	51	69	12	498	527	437	0.83 (0.50;1.38)	1.07 (0.74;1.54)	0.80 (0.39;1.62)	
Per 50 g/day							0.82 (0.63;1.07)	1.12 (0.91;1.37)	0.91 (0.59;1.39)	0.28
Poultry ^6^										
Low	63	125	37	811	930	711	1.00 Reference	1.00 Reference	1.00 Reference	0.88
Medium	77	135	65	563	501	539	1.24 (0.88;1.75)	0.89 (0.69;1.14)	1.03 (0.68;1.54)	
High	32	71	35	418	527	583	0.87 (0.56;1.36)	0.95 (0.71;1.28)	0.96 (0.60;1.55)	
Per 100 g/day							0.93 (0.48;1.81)	0.77 (0.47;1.26)	1.41 (0.72;2.78)	0.21

Abbreviations: n, number of participants; IR, incidence rates; HR, hazard ratios; CI, confidence interval. ^1^ Low compliance < 2.4 on the Dietary Guideline Compliance Score; Medium compliance 2.4–3.7 on the Dietary Guideline Compliance Score; high compliance > 3.7 on the Dietary Guideline Compliance Score. Dietary Guideline Compliance Score expresses the dietary compliance with the five quantitative Danish dietary guidelines on fruit and vegetables, fish, whole grain, saturated fatty acids, and added sugars. It can vary between 0 and 5. ^2^ Per 100,000 person-years. ^3^ Adjusted by sex, age, educational attainment, ethnicity, smoking, physical activity, alcohol, BMI, and total energy intake. ^4^ Red meat intake: low < 41 g/day; medium 41–97 g/day; high > 97 g/day. ^5^ Processed meat intake: low < 19 g/day; medium 19–58 g/day; high > 58 g/day. ^6^ Poultry intake: low < 1 g/day; medium 1–34 g/day; high > 34 g/day.

## Data Availability

Due to Danish law, the confidential health care data used in this study can only be accessed through the servers of Statistics Denmark. Access is granted upon request if the applicant fulfils the criteria for access. Statistics Denmark can be contacted by e-mail: databanker@dst.dk.

## Supplementary Materials

The following are available online at https://www.mdpi.com/2072-6643/13/1/32/s1, Table S1: Baseline characteristics of the colorectal cancer study population stratified by dietary guideline compliance and intake of processed meat; Table S2: Baseline characteristics of the colorectal cancer study population stratified by dietary guideline compliance and intake of poultry.
