# Supplementary material for: Dietary Intake of Red Meat, Processed Meat, and Poultry and Risk of Colorectal Cancer and All-Cause Mortality in the Context of Dietary Guideline Compliance
Source: Nutrients. 2020 Dec 23;13(1):32. doi: 10.3390/nu13010032 (PMC7823645; doi:10.3390/nu13010032)
Supplement: Supplementary file 1 [file nutrients-13-00032-s001.pdf]

Dietary intake of red meat, processed meat, and poultry and risk of colorectal cancer and all-cause mortality in the context of dietary guideline compliance

Supplement tables

Table S1. Baseline characteristics of the colorectal cancer study population stratified by dietary guideline compliance and intake of processed meat, n=6,282

**Dietary guideline compliance[1]**	**Low**		**High**	
**Processed meat intake[2]**	**Low**	**High**	**Low**	**High**
Age, mean (SD)	53 (11)	52 (11)	56 (10)	55 (10)
	**n (%)**	**n (%)**	**n (%)**	**n (%)**
Sex				
Men	433 (38.5)	1233 (72.0)	553 (26.7)	814 (59.5)
Women	693 (61.5)	480 (28.0)	1522 (73.3)	554 (40.5)
Ethnicity				
Danish	1089 (96.7)	1691 (98.7)	2007 (96.7)	1341 (98.0)
Western	16 (1.4)	15 (0.9)	33 (1.6)	15 (1.1)
Non-western	21 (1.9)	7 (0.4)	35 (1.7)	12 (0.9)
Educational level[3]				
Long	286 (25.4)	379 (22.1)	797 (38.4)	465 (34.0)
Medium	500 (44.4)	836 (48.8)	734 (35.4)	595 (43.5)
Short	340 (30.2)	498 (29.1)	544 (26.2)	308 (22.5)
BMI				
Underweight	26 (2.3)	21 (1.2)	34 (1.6)	10 (0.7)
Normal weight	600 (53.3)	753 (44.0)	1133 (54.6)	635 (46.4)
Overweight	371 (32.9)	682 (39.8)	676 (32.6)	522 (38.2)
Obese	129 (11.5)	257 (15.0)	232 (11.2)	201 (14.7)
Smoking				
Never	384 (34.1)	611 (35.7)	988 (47.6)	615 (45.0)
Former	285 (25.3)	473 (27.6)	688 (33.2)	513 (37.5)
Current	457 (40.6)	629 (36.7)	399 (19.2)	240 (17.5)
Leisure time physical activity				
None	149 (13.2)	176 (10.3)	124 (6.0)	71 (5.2)
Light	514 (45.6)	704 (41.1)	822 (39.6)	522 (38.2)
Moderate/hard	463 (41.1)	833 (48.6)	1129 (54.4)	775 (56.7)

Abbreviations: n, number of participants; SD, standard deviation; BMI, Body Mass Index

[1]Low compliance < 3.1 on the Dietary Guideline Compliance Score; High compliance ≥ 3.1 on the Dietary Guideline Compliance Score. Dietary Guideline Compliance Score express the dietary compliance with the five quantitative Danish dietary guidelines on fruit and vegetables, fish, whole grain, saturated fatty acids and added sugars. It can vary between 0 and 5

[2]Processed meat intake: Low < 35 g/day; High ≥ 35 g/day

[3]Long: higher education, medium: high school or vocational school, short: primary school

Table S2. Baseline characteristics of the colorectal cancer study population stratified by dietary guideline compliance and intake of poultry, n=6,282

**Dietary guideline compliance**[1]	**Low**		**High**	
**Poultry intake**[2]	**Low**	**High**	**Low**	**High**
Age, mean (SD)	53 (11)	51 (11)	56 (10)	54 (10)
	**n (%)**	**n (%)**	**n (%)**	**n (%)**
Sex				
Men	855 (57.2)	811 (60.4)	676 (39.5)	691 (39.9)
Women	641 (42.8)	532 (39.6)	1035 (60.5)	1041 (60.1)
Ethnicity				
Danish	1468 (98.1)	1312 (97.7)	1667 (97.4)	1681 (97.1)
Western	17 (1.1)	14 (1.0)	20 (1.2)	28 (1.6)
Non-western	11 (0.7)	17 (1.3)	24 (1.4)	23 (1.3)
Educational level[3]				
Long	334 (22.3)	331 (24.6)	612 (35.8)	650 (37.5)
Medium	713 (47.7)	623 (46.4)	647 (37.8)	682 (39.4)
Short	449 (30.0)	389 (29.0)	452 (26.4)	400 (23.1)
BMI				
Underweight	25 (1.7)	22 (1.6)	27 (1.6)	17 (1.0)
Normal weight	742 (49.6)	611 (45.5)	868 (50.7)	900 (52.0)
Overweight	529 (35.4)	524 (39.0)	593 (34.7)	605 (34.9)
Obese	200 (13.4)	186 (13.8)	223 (13.0)	210 (12.1)
Smoking				
Never	511 (34.2)	484 (36.0)	786 (45.9)	817 (47.2)
Former	388 (25.9)	370 (27.6)	594 (34.7)	607 (35.0)
Current	597 (39.9)	489 (36.4)	331 (19.3)	308 (17.8)
Leisure time physical activity				
None	189 (12.6)	136 (10.1)	102 (6.0)	93 (5.4)
Light	653 (43.6)	565 (42.1)	672 (39.3)	672 (38.8)
Moderate/hard	654 (43.7)	642 (47.8)	937 (54.8)	967 (55.8)

Abbreviations: n, number of participants; SD, standard deviation; BMI, Body Mass Index

[1]Low compliance < 3.1 on the Dietary Guideline Compliance Score; High compliance ≥ 3.1 on the Dietary Guideline Compliance Score. Dietary Guideline Compliance Score express the dietary compliance with the five quantitative Danish dietary guidelines on fruit and vegetables, fish, whole grain, saturated fatty acids and added sugars. It can vary between 0 and 5

[2]Poultry intake: Low < 16 g/day; High ≥ 16 g/day

[3]Long: higher education, medium: high school or vocational school, short: primary school
